# Screening of selected ethnomedicinal plants from South Africa for larvicidal activity against the mosquito *Anopheles arabiensis*

**DOI:** 10.1186/1475-2875-11-320

**Published:** 2012-09-10

**Authors:** Rajendra Maharaj, Vinesh Maharaj, Neil R Crouch, Niresh Bhagwandin, Peter I Folb, Pamisha Pillay, Reshma Gayaram

**Affiliations:** 1 South African Medical Research Council, 491 Ridge Road, Overport, Durban, 4001, South Africa; 2 Biosciences, CSIR, P.O.Box 395, Pretoria, 0002, South Africa; 3 Ethnobotany Unit, South African National Biodiversity Institute, School of Chemistry and Physics, University of KwaZulu-Natal, PO Box 52099, Berea Road 4007, Durban, 4041, South Africa; 4 South African Medical Research Council, P.O.Box19070, Tygerberg, 7505, South Africa

## Abstract

**Background:**

This study was initiated to establish whether any South African ethnomedicinal plants (indigenous or exotic), that have been reported to be used traditionally to repel or kill mosquitoes, exhibit effective mosquito larvicidal properties.

**Methods:**

Extracts of a selection of plant taxa sourced in South Africa were tested for larvicidal properties in an applicable assay. Thirty 3^rd^ instar *Anopheles arabiensis* larvae were exposed to various extract types (dichloromethane, dichloromethane/methanol) (1:1), methanol and purified water) of each species investigated. Mortality was evaluated relative to the positive control Temephos (Mostop; Agrivo), an effective emulsifiable concentrate larvicide.

**Results:**

Preliminary screening of crude extracts revealed substantial variation in toxicity with 24 of the 381 samples displaying 100% larval mortality within the seven day exposure period. Four of the high activity plants were selected and subjected to bioassay guided fractionation. The results of the testing of the fractions generated identified one fraction of the plant, *Toddalia asiatica* as being very potent against the *An. arabiensis* larvae.

**Conclusion:**

The present study has successfully identified a plant with superior larvicidal activity at both the crude and semi pure fractions generated through bio-assay guided fractionation. These results have initiated further research into isolating the active compound and developing a malaria vector control tool.

## Background

Malaria remains one of the highest priority insect transmitted diseases around the world, with Africa carrying the greatest burden. The *Anopheles arabiensis* mosquito is considered one of the major vectors of malaria in southern Africa with *Anopheles funestus* the next most important [1].

Within South Africa, malaria affects five million people in the low and high risk areas of the country [2] with the threat concentrated in Mpumalanga, Limpopo and (north-eastern) KwaZulu Natal provinces [3]. According to the strategic plan for communicable diseases in South Africa, one of the main objectives is addressing malaria, to reduce the incidence of local transmission from 0.7 to 0.56 cases per thousand. Strategies employed include indoor spraying, definitive diagnosis of malaria cases and effective case management [4]. The malaria control strategy in South Africa has a two-pronged approach, targeting the malaria parasite with anti-malarial drugs and controlling the vector through the use of insecticides, targeting both larval and adult life stages [5].

Historically, the use of synthetic insecticides has been very effective in reducing malaria transmission. However, over time success has been hampered by the development of insecticide resistance in mosquitoes. Resistance to pyrethroids [6] and DDT [7] has been reported, and the potential for carbamate resistance has been detected in *An. arabiensis,* in northern Kwazulu-Natal [8].

The effective alternative insecticides that are currently available are up to six times more expensive than those (DDT and pyrethroids) used previously in regional malaria control programmes and accordingly are not cost effective for indoor residual spraying [9].

As most mosquito breeding sites are temporary habitats, the use of larvicides by malaria control programmes has been very limited. Larvicidal applications have however been favoured mainly in tourist destinations to limit mosquito populations. This form of vector control has been used as a complementary strategy to indoor residual spraying (for adult stages) and has been suggested as a method for eliminating over-wintering larval populations [10,11].

Since insecticide resistance threatens to contribute towards the reintroduction of malaria in many parts of South Africa, efforts have focussed on finding an alternative form of mosquito control. For new interventions to be integrated into most malaria control programmes, they should necessarily be cost effective, practical and accessible.

The concept of screening plant extracts for larvicidal activity is not new. The insecticide pyrethroid was derived from flowers of the asteraceous *Tanacetum cinerariifolium* (= *Chrysanthemum cinerariifolium*, the Dalmatian chrysanthemum or pyrethrum daisy) [12]. Production of such constituents has been interpreted as a means by which plants protect themselves from harmful insect herbivores. The notion of using such plant extracts to control mosquito larvae has received widespread research attention, with plants from various countries tested for larvicidal activity [13-16]. Such plant extracts have been investigated for their ability to kill a number of mosquito species known to be malaria vectors [14,17-19].

The efficacy of different plant parts has been well established [16,20,21]. The flower [22], leaf and seed [21], fruits and roots [23,24] have all been investigated for their larvicidal properties, revealing variably useful leads for the development of practical interventions.

Although a small number of studies have investigated larvicidal activity of plant extracts against *Anopheles stephensi*[25,26] there are limited data regarding the testing of plant extracts against *An. arabiensis*. Zarroug *et al.*[27] tested some Sudanese plant extracts against *An. arabiensis* larvae and obtained significant mortality in second and fourth instar larvae when using water extracts of *Balanites aegyptiaca*.

In the present study, 381 crude plant extracts were investigated for their larvicidal effect on *An. arabiensis*, and plants exhibiting promising results during screening were selected for further phytochemical investigation. The extracts consisted of plants found native or naturalized in southern Africa.

## Methods

### Selection and collection of plant material

A survey of relevant published literature on ethnomedicinal plants used in East and southern Africa revealed that a number of taxa have been reported to be used as mosquito repellents, or to repel or kill other invertebrates. However, given the limited quantity of documented data available, it was decided to not make a distinction between insect repellents and insecticides (both larva- and adulticides), but rather to consider a pool of plants with anti-insect activity.

In order to select the most relevant taxa for screening, all were ranked following the application of weighted criteria, principally ethnobotanical and chemotaxonomic (including such elements as popularity in ethnomedicinal trade, reports on insecticidal and/or mosquitocidal application, reports on insect and/or mosquito repellent application, and the known presence and diversity of repellent/insecticidal constituents in the family to which it belongs). Higher weighting was provided to plants indigenous to the Flora of Southern Africa region (FSA). A similar semi-quantitative selection method has previously been applied to identify and rank both anti-plasmodial [28], mosquito repellent [29] and adulticidal plant [30] candidates from South Africa. From the ranked list selected plants were collected throughout South Africa. Different plant parts, namely, leaves, roots, stems, fruits, flowers, seeds, twigs and bark, and combinations of the above were sourced to generate extracts. In some instances, extracts were made of the whole plant. The plant organ(s) selected for extract preparation were based largely on availability at the time of collection.

The identity of plant material was determined at the National Herbarium of South Africa (PRE) where voucher specimens (cited in Additional file 1: Table S1) have been lodged.

### Extract preparation

Plant samples were separated into different components and dried in an oven at 30–60°C. The drying time and temperature varied depending on the nature of the plant part. Dried plant material was ground to a coarse powder using a hammer mill and stored at ambient temperature prior to extraction. For each extraction procedure, 100–500 g of powdered plant material was sequentially extracted, with cold dichloromethane (DCM), DCM/methanol (MeOH) (1:1), MeOH (CP Grade; Merck) and purified water. Organic extracts were concentrated by rotary vacuum evaporation below 45°C and then further dried *in vacuo* at ambient temperature for 24 h. The aqueous extracts were concentrated by freeze-drying. All dried extracts were stored at −20°C.

Crude plant samples were dissolved in either acetone (AR Grade; Merck) or distilled water depending on their initial extraction procedure, thus forming a 10 mg/ml solution. Dichloromethane and dichloromethane/methanol extracts were reconstituted in acetone whereas aqueous extracts were made up using distilled water.

### Larvicidal screening

A 1 ml volume of the extract stock solution was added to a vessel containing thirty 3^rd^ instar mosquito larvae in 0.25 litres of distilled water, producing a final concentration of 40 mg/l. The target species had been a colonized strain of *An. arabiensis* from Zimbabwe, which had been reared according to the World Health Organization’s [31] guidelines in an insectary simulating the temperature, humidity and lighting of a malaria endemic environment. The negative control trials included either acetone or distilled water, whilst the positive control temephos (Mostop; Agrivo), an effective larvicide was tested at a concentration of 0.2 mg/l.

Each container was monitored for larval mortality at 24-hour intervals for a period of seven days and fed at regular intervals. The percentage mortality was calculated relative to the negative control.

## Results and discussion

A total of 80 taxa from 42 families of plants native to or naturalized in southern Africa have been screened for activity against the 3^rd^ instar larvae of *An. arabiensis*. The third stage of larvae had been used to determine if the extracts had induced any growth inhibition or abnormalities in ecdysis to 4^th^ instar and pupation. Significant events had included failed or delayed transition relative to negative control. The results of primary screening have been presented alphabetically by family, genus and species and thereafter in descending order of mortality over a period of seven days (Cited in Additional file 1: Table S1). In order to assess the biological activity of each extract, all samples were subjected to stringent criteria to identify plants that exhibited larvicidal properties. Any plant with activity of more than 80% mortality was considered significant for further investigation [32]. However, it must be borne in mind that assays were conducted primarily using the crude plant extract.

The results of preliminary screening of 381 plant extracts against the third instar larvae revealed substantial variation in toxicity, differing with plant species, organ, and extraction type. Whilst a substantial number of plants showed moderate to low toxic effects, almost 11% (24 extracts) of the total samples possessed good larvicidal properties relative to the positive control. In an earlier study, Maharaj *et al.*[30] screened crude extracts of many of the same plant species here reported on, for adulticidal properties. Very limited adulticidal activities were observed, indicating a much greater susceptibility of larvae to the same extracts. The toxicity of the highly active positive control sample had exhibited 100% mortality within 24 hours of exposure, whilst both negative control tests indicated insignificant toxicity of solvents used.

Of the 42 extracts exhibiting high activity in this study, a total of 24 samples demonstrated excellent results (100% mortality during the seven day exposure period) (Table 1). In a separate study, the crude extracts used in this experiment were screened and five samples with repellent properties identified during preliminary assays [29]. Although none of the potential repellents had maintained a repellent effect over a prolonged period, one of five samples, *Litogyne gariepina* had also been identified as a potential larvicide in the current study, suggesting a dual effect in different environments.

**Table 1:** Crude extracts exhibiting 100% mortality during preliminary laboratory trials

**Family**	**Plant species**	**Voucher number**	**Plant part**	**Extraction**	**Mortality time (hours)**
Araliaceae	*Cussonia spicata* Thunb.	EN00867	Fruit	DCM	72
Asphodeleace	*Aloe greatheadii* Schönland var. *davyana* (Schönland) Glen & D.S.Hardy	EN00021	Leaves and twigs	DCM	120
Asteraceae	*Litogyne gariepina* (DC.) Anderb.	EN00213	Roots	DCM	96
	*Pentzia globosa* Less.	EN00506	Leaves	Water	48
	*Psiadia punctulata* (DC.) Oliv. & Hiern ex Vatke	BP00278	Leaves	DCM	144
	*Vernonia natalensis* Oliv. & Hiern	EN00331	Whole plant	Water	48
Capparaceae	*Capparis tomentosa* Lam.	EN00222	Stems	Water	24
	*Capparis tomentosa* Lam.	EN00222	Leaves	Water	48
Ebenaceae	*Euclea natalensis* A.DC.	EN00760	Roots	DCM	48
	*Euclea natalensis* A.DC.	EN00760	Stems	DCM	96
Euphorbiaceae	*Ricinus communis L.	EN00768	Fruit	DCM	48
Fabaceae	*Philenoptera violacea* (Klotzsch) Schrire	MM00019	Stems	DCM	24
	*Pterocarpus angolensis* DC.	EN00083	Roots	Water	48
	*Pterocarpus angolensis* DC.	EN00083	Roots	MeOH	144
Lamiaceae	*Hyptis pectinata (L.) Poit.	BP00243	Leaves, stems and fruits	Water	48
Olacaceae	*Ximenia caffra* Sond. var. ca*ffra*	EN00110	Roots	Water	48
Phyllanthaceae	*Flueggea virosa* (Roxb. ex Willd.) Voigt	BP00207	Leaves and twigs	Water	24
Plumbaginaceae	*Plumbago zeylanica* L. (1)	EN00208	Leaves	DCM	48
	*Plumbago zeylanica* L. (2)	EN00208	Leaves	DCM	48
Rutaceae	*Macrostylis squarrosa* Bartl. & H.L.Wendl.	EN00758	Stems	DCM	24
	*Toddalia asiatica* (L.) Lam. (1)	EN00211	Leaves	DCM	72
	*Toddalia asiatica* (L.) Lam. (2)	EN00211	Leaves	DCM	72
Thymelaeaceae	*Gnidia cuneata* Meisn.	EN00716	Stems	DCM	144
Zingiberaceae	*Siphonochilus aethiopicus* (Schweinf.) B.L.Burtt	EN00148	Rhizomes	Ether	72

*exotic to South Africa.

Results indicated that organic extracts exhibit a greater effect than aqueous extracts, a trend also noted in the previous repellency study [29]. In another similar insecticidal experiment, two of the highly active families screened during this experiment, Rutaceae and Zingiberaceae, were reported to yield promising botanical larvicides [33]. It was also observed in this study that almost half of the prioritized extracts induced mortality within 48 hours, suggesting a very high toxic effect at the crude stage.

Based on the mortality obtained from a large number of crude extracts, the biological activity of each plant was assessed. The most promising candidates represented by four taxa and four extracts were subjected to bioassay-guided fractionation. A total of 36 semi pure fractions were tested in a manner similar to that of the crude extracts (Table 2). A loss of activity was observed in two of the four extracts, relative to the preliminary screening of crude samples. *Aloe greatheadii* var. *davyana* (Asphodelaceae) showed 100% mortality for only one fraction, whilst four of the five fractions of *Toddalia asiatica* (Rutaceae) induced 100% mortality within 96 hours of exposure, making it the favoured extract for further investigation of active compounds. The Rutaceace have earlier been shown to induce insecticidal effects against mosquitoes [34,35]. *Murraya koenigii* possesses mosquitocidal properties through effects of hormone regulation with subsequent disruption of instar development of *Anopheles stephensi*, *Culex quinquefasciatus* and *Aedes aegypti*[34]. Tiwari *et al.*[35] found that the essential oil obtained from seeds of *Zanthoxylum armatum* (Rutaceae) displayed promising larvicidal activity when tested against *An. stephensi*, *Cx quinquefasciatus* and *Ae. aegypti* larvae.

**Table 2:** The larvicidal activity of thirty six fractions of the most promising plant extracts

**Plant species**	**Family**	**Plant part**	**Extraction**	**Fraction Number**	**% Mortality**
Rutaceae	*Macrostylis squarrosa*	Stems	DCM	1-7	22
				11-14	70
				19-21	68
				22	25
				23	5
				24	18
				26-29	57
				44-63/17-20	77
Rutaceae	*Toddalia asiatica*	leaves	DCM	2-3	10
				100-122	100
				17-27/36-60	100
				36-47/8-12	100
				168-180/11-19	100
Asphodeleace	*Aloe greatheadii* var. *davyana*	Leaves + Twigs	DCM	1-11	18
				12-18	43
				19-27	23
				28-32	32
				33-41	100
				42-52	20
				53-54	17
				55-67	58
				68-75	42
				76-77	63
				78-82	25
				83-85	12
Asteraceae	*Vernonia natalensis*	All parts	Water	31-35	27
				36-38	10
				29-30	22
				1-28	20
				39-41	32
				42-44	40
				45-50	28
				51-54	12
				55-65	50
				66-69	57
				70-76	45

*Toddalia asiatica* has gained popularity amongst traditional health practitioners for treating numerous ailments. Amongst the documented ethnomedicinal uses, the fruit of this plant is known to have been popularly applied in treating malaria, particularly in East Africa [36]. The current study has revealed that the leaves of *T. asiatica* contain compounds toxic against the larvae of *An. arabiensis*. The current findings compare well with a study conducted in India, which has revealed that *T. asiatica* fruits contains anti-larval compounds active against the 3^rd^ instar stages of the two mosquito vectors, *Ae. aegypti* and *Cx quinquefasciatus*[37].

## Conclusions

Although organophosphates, such as temephos, have shown to be very effective in this study, they are not favoured due to their high toxicity to humans, low stability and high cost implications [38]. Bioactive constituents of plants hold potential to be employed as larvicides useful in controlling mosquito vectors [39]. The present study has successfully identified a plant with superior larvicidal activity for both the crude and semi pure fractions thus indicating a potential for use in the control of the malaria vector, *An. arabiensis*. Results obtained during this study have initiated ongoing investigations into the isolation of purified active components of *T. asiatica*, with a view to developing an environmentally acceptable tool of value in integrated vector control.

## Competing interests

There have been no competing interests.

## Authors' contributions

RM was involved in designing of the study and supervising trials. RG conducted the experiments and was involved in the interpretation of the results. NRC was involved in rationally selecting suitable plant candidates for investigation. Extracts were prepared by VM and PP. NB and PF coordinated and provided scientific inputs into the entire study. All authors read and approved the final manuscript.

## Supplementary Material

Additional file 1 Table S1. Mosquito larvicidal screening results of extracts from South African ethnomedicinal plants. Click here for file
